# Spot the difference: Turbidity‐driven plasticity and variation in spotted grunter (*Pomadasys commersonnii*) spot patterns

**DOI:** 10.1111/jfb.70646

**Published:** 2026-09-10

**Authors:** Vivienne Coetzee, Denham Parker, Stephen Lamberth, Sven Kerwath

**Affiliations:** ^1^ Department of Biological Sciences University of Cape Town Rondebosch South Africa; ^2^ WILDTRUST Pietermaritzburg South Africa; ^3^ CSIRO Environment Brisbane Queensland Australia; ^4^ Department of Forestry, Fisheries and Environment Cape Town South Africa; ^5^ Institute for Coastal and Marine Research, Ocean Sciences Campus Nelson Mandela University Gqeberha South Africa

**Keywords:** morphological colour change, phenotypic plasticity, photo‐identification, sensory drive hypothesis

## Abstract

Animal body markings such as spots, stripes and patterns play important roles in camouflage and visual communication. This study investigated phenotypic plasticity in the spot patterns of spotted grunter *Pomadasys commersonnii*, an estuarine‐dependent haemulid species. First, an aquarium experiment (*n* = 24) examined plasticity in spot patterns under contrasting turbidity conditions. *P. commersonnii* reduced their spot size rapidly and reversibly in response to elevated turbidity, with spots returning close to baseline once clear less turbid conditions were restored, whereas the number and position of spots remained constant throughout the experiment, suggesting that spot patterns may be useful for short‐term individual identification. Second, spot patterns of *P. commersonnii* (*n* = 139) from two estuaries with contrasting turbidities were compared. After accounting for body size, fish from the less turbid Breede Estuary had more numerous spots than those from the turbid Kei Estuary and accumulated spots more rapidly with growth, whereas spot size did not differ between estuaries. Spots from the Kei Estuary were, however, more elongated and differently orientated and individuals could be assigned to their estuary of origin with 83.5% accuracy on the bases of spot patterning alone. These findings are consistent with the sensory drive hypothesis, whereby individuals adapt their communication systems to local environmental conditions and indicates that spot size responds plastically to current turbidity while spot number reflected a longer‐term, developmentally accumulated difference between estuaries. Although the precise function of these spots remains unknown, environmental variables such as turbidity evidently play an important role in the phenotypic variation of *P. commersonnii*.

## 1 INTRODUCTION

Animals possess a wide diversity of colourful‐patterned skins, integuments, pelages and plumages which can be so pronounced that species are named after them, such as the spotted grunter, *Pomadasys commersonnii*. These visual traits typically serve one of two functions: camouflage and communication. The ability to blend into the surrounding environment reduces the likelihood of detection and recognition by predators or competitors, which is advantageous to survival (Duarte et al., 2017). Animals employ various forms of camouflage, most notably background matching, when an animal's body colouration is optimized to resemble the surrounding habitat to reduce detection by visual predators (Michalis et al., 2017) and disruptive colouration, which confuses observers by creating false boundaries and disrupting an animal's silhouette (Cott, 1940; Thayer, 1918). The effectiveness of camouflage depends on the visual environment, with light conditions playing a crucial role in predator detection (Endler, 1993).

Alternatively, visual signals can transmit information between individuals (Morrongiello et al., 2010). Aposematic colouration, for example, advertises an individual's toxicity, with some non‐toxic species evolving Batesian mimicry to exploit these widely understood visual signals (Briolat et al., 2019). Beyond predator–prey interactions, patterns can also express the quality or fitness of an individual and are thus important for both sexual selection and intra‐specific competition. For example, female three‐spine sticklebacks (*Gasterosteus aculeatus*) prefer males exhibiting bright nuptial displays with blue eyes, bright red throats and anterior bellies during breeding season (Smith et al., 2004).

Many fish species display ontogenetic colour change, or polyphenism, whereby colouration shifts between juvenile to adult stages (Figon & Casas, 2018). This is common in species that transition between habitats as they mature, functioning primarily as adaptive camouflage. However, ontogenetic colour changes can also occur in species that remain resident throughout their life, signalling sexual maturity or reducing adult male aggression towards juveniles during breeding season. In the emperor angel fish (*Pomacanthus imperator*), juveniles display distinct colouration that prevents adults from perceiving them as competitors (Fricke, 1980).

Body colouration is associated with adaptation, and the capacity for this trait to be plastic is essential for many species (Sköld et al., 2016). Phenotypic plasticity provides a faster, more diverse response to environmental change than genetic adaptation alone (West‐Eberhard, 2003) and according to the sensory drive hypothesis, individuals may alter their signalling systems to suit local environmental conditions (Boughman, 2002; Endler, 1992). Plasticity is therefore particularly advantageous for species inhabiting a wide range of environments (Price, 2006). Plasticity in colour patterns is beneficial when balancing the many selective pressures that colour patterns undergo, and these changes can occur under two mechanisms. Morphological colour change which happens over weeks to months and involves either changing the number and proportion of chromatophores (Sköld et al., 2016), or chemical modification and degradation of cell pigment (Figon & Casas, 2018). Whereas physiological colour change is rapid, occurring over seconds to minutes and involves the aggregation of pigments cells within chromatophores (Fujii, 2000). For example, Japanese rice fish (*Oryzias latipes*) increase their leucophore (white pigment) density and lower their melanophore (black/brown pigment) and xanthophores (yellow/orange pigment) when exposed to white backgrounds (Sugimoto, 2002), demonstrating morphological colour change to optimize their camouflage. The distinction matters because physiological changes are typically rapid and reversible, whereas morphological changes involve structural reorganization and are generally slower but longer‐lasting (Sköld et al., 2013).

A trait needs to provide a certain level of fitness to be evolutionarily stable, but these benefits may be counteracted by the energetic costs needed to sustain these traits (DeWitt et al., 1998; Sugimoto, 2002). The costs associated with plasticity include the energetic demands of maintaining regulatory mechanisms, producing and reorganizing pigment cells, and trade‐offs in resource allocation to other fitness‐related traits (Auld et al., 2010). Sculpins (Family Cottidae) exhibit reduced survival rates following colour change (Bergstrom et al., 2012), while guppies (Family Poeciliidae) increase their food consumption in response to repeated colour changes (Rodgers et al., 2013). While much research still needs to be conducted on the costs involved with colour change, the consensus is that the cost incurred in each scenario is dependent on the speed and type of colour change.

Turbidity is a key factor affecting the visual environment by reducing the quantity and quality of light, altering the spectral composition available for visual signalling, which can greatly influence colour pattern development and expression (Endler, 1993; Gray et al., 2011). By decreasing ambient light and shifting the environment towards darker conditions, turbidity can compromise the efficacy of visual signals, since signal detection depends on the receiver's ability to detect it (Van der Sluijs et al., 2011). Several fish species exhibit plasticity in their nuptial colouration when exposed to different environmental conditions. Male pygmy perch (*Nannoperca australia*) maximize their nuptial signal efficacy in light environments by increasing the intensity of their red colouration (Morrongiello et al., 2010), while male guppies living in turbid environments have a greater degree of structural colour compared to those in clearer waters (Adro'i et al., 2018). Water clarity and visibility also affect the relationship between species diversity and colour diversity (Seehausen et al., 1997). Male cichlid fish in clear waters within Lake Victoria have distinct, bright colourations compared to those living in areas with poor visibility (Seehausen et al., 1997). Notably, *Pundamilia nyererei*, a species of cichlids in Lake Victoria, were found to have duller red colouration and reduced standards for mate choice when inhabiting turbid waters compared to those in clear water environments. Thus, turbidity degrades image quality and plays an important role in signalling and communication (Seehausen et al., 1997; Utne‐Palm, 2002).

Spotted grunter, characterized by pronounced black spots along their light grey body, are an estuarine‐dependent, western Indian Ocean coastal species ranging from India to False Bay in South Africa (Heemstra & Heemstra, 2004). Being eurytopic, they can occupy a wide range of estuarine and coastal conditions (Whitfield, 2005), and this tolerance of variable environments makes rapid adjustment of body patterning to local conditions potentially advantageous. In South Africa, spotted grunter are currently managed as a single stock Næsje et al., 2007. Historically, spawning was thought to occur exclusively in the coastal waters of KwaZulu‐Natal, but over recent decades the species has established in the Western Cape (Augustyn et al., 2017). Year‐round residency and spawning have since been documented in the Breede and Knysna estuaries (Augustyn et al., 2017; Butler et al., 2022 ; Kerwath et al., 2005), and telemetry indicates that Western Cape fish are largely resident, undertaking only short sea excursions rather than long distance migrations (Ziko et al., 2023).

It is assumed that spotted grunter spots function in either camouflage or visual communication, though their role in visual signalling remains uncertain. If spots aid camouflage through background matching, higher spot densities would be expected in visually complex, heterogeneous environments (Kelley et al., 2013). Conversely, in homogeneous turbid environments, fewer spots may better conceal an individual against a featureless background. If instead spots function in communication, fewer spots would again be expected in turbid environments, where low signal transmission may not justify the cost of producing them. Producing and maintaining conspicuous spot patterns is thought to carry energetic costs, so in turbid environments, where visual communication is reduced, selection or plasticity may favour their reduction.

This study had two aims: (1) to investigate whether spotted grunter exhibit phenotypic plasticity in their spot patterns when exposed to different turbidity levels, using a controlled aquarium experiment and (2) to compare the spot patterns of spotted grunter from two estuaries with contrasting turbidities, the turbid Kei estuary in the Eastern Cape and the clearer Breede Estuary in the Western Cape of South Africa (James et al., 2018; Nhleko, 2025). The two systems differ markedly in turbidity, with average turbidity in the Kei recorded at approximately 1300 NTU compared to 7.4 NTU in the Breede (Harrison, 2004). To exclude confounding factors due to the difference in the biogeography of the two estuaries, the aquarium experiment was designed to test the effect of turbidity directly, under conditions where other environmental variables were held constant. For the experiment, we hypothesized that increased turbidity would result in fewer, smaller spots (Figure 1).

**FIGURE 1:**
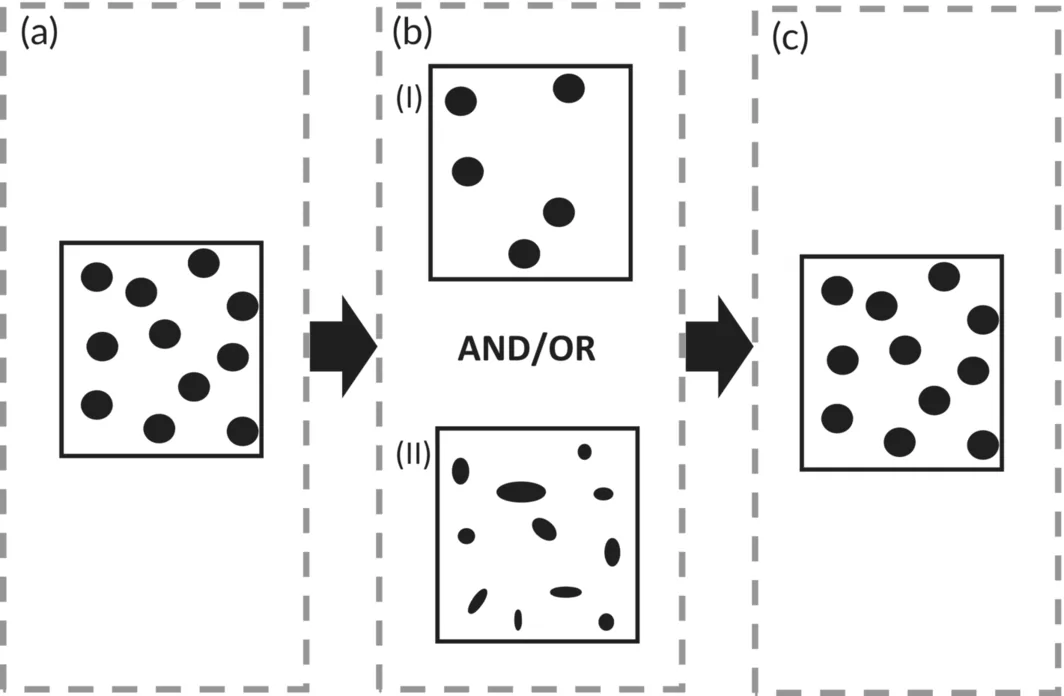
Spot pattern plasticity infographic: Diagram indicating the predictions of spot pattern changes in *Pomadasys commersonnii* when exposed to varying levels of turbidity. (a) The hypothetical spot pattern of a *P. commersonnii* at the start of the experiment. (b) The predicted response in spot patterns of *P. commersonnii* when exposed to increased levels of turbidity: (I) fewer spots and (II) a change in spot size/shape. (c) The expected outcome of the experiment when returned to low turbidity environments, that is, reversal in spot patterns.

## 2 METHODS

### 2.1 Ethics statement

All procedures in this study were approved by the Department of Environment, Forestry and Fisheries (RES2020/86, RES2021/56).

### 2.2 Experiment 1: Phenotypic plasticity in spot patterns

#### 2.2.1 Experimental design

Twenty‐four F2 captive‐bred spotted grunter were obtained from aquarium stock; husbandry prior to the experiment followed standard facility protocols. Fish were divided among four 4000 L tanks (*n* = 6 per tank) arranged in a 2 × 2 grid. Before the experiment, all tanks were connected to the same filtration system, ensuring uniform water variables including temperature, salinity and dissolved oxygen levels. At the start of the experiment, the tanks were isolated from the system and maintained as closed systems. To limit the accumulation of waste and toxins in the closed tanks, fish were not fed during the treatment portion of the experiment. The short treatment duration was chosen to minimize deterioration of water quality in the absence of active filters; routine checks were carried out by facility staff throughout this period. Each tank contained two small (<30 mm), two medium (30–50 mm) and two large [>50 mm total length (TL)] spotted grunter to maintain similar size distributions.

Two tanks were randomly assigned to each treatment: clear (control <50 NTU) and turbid (>100 NTU; Figure S1). Turbidity was created using bentonite clay at the start of the experiment (Benfield & Minello, 1996; Madsen et al., 1999; Pusch, 2015) and kept in suspension by a circulation pump in each tank to prevent the sediment from settling (Figure S1c).

The fish were photographed every 4 days over the 12‐day period. To capture the fish, as per our ethics approval, 2‐phenoxyethanol was used to sedate the fish (Deacon & Hecht, 1996). Once sedated, each fish was caught, measured and photographed (Huawei P30 Pro, Leica optics camera) outside of the water on a black background. Once the 12‐day experiment was completed, the fish were returned to the standard aquarium filtration system for 3 weeks before final photographs were taken to assess the reversibility of spot patterns.

#### 2.2.2 Spot image analysis

Spot patterns were analysed using ImageJ, an open‐source, Java‐based program specifically designed for image processing and analysis in the scientific field (Schneider et al., 2012). Each image is analysed independently from start to finish (Figure 2).

**FIGURE 2:**
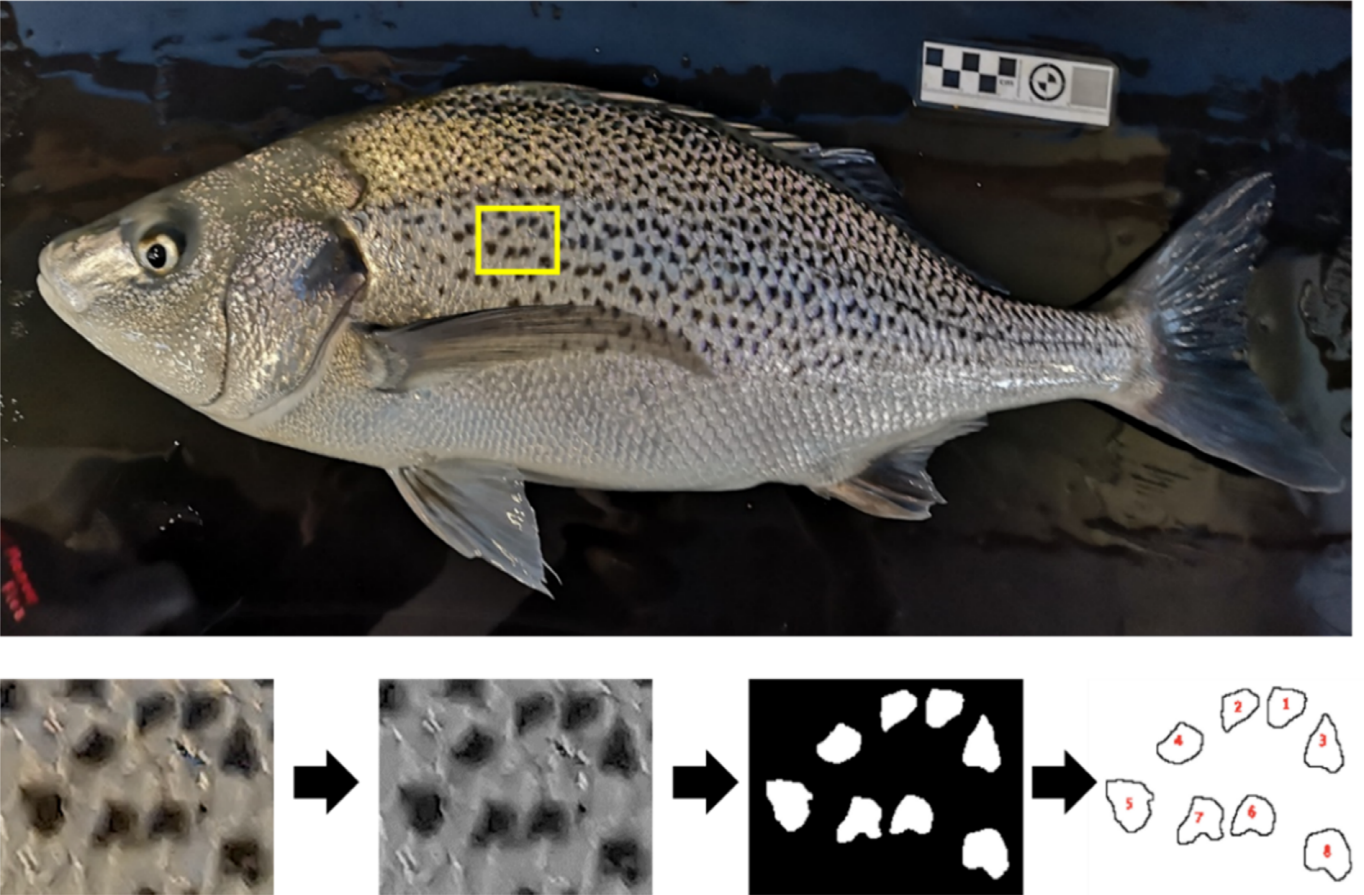
ImageJ spot analysis: (Top) Sedated fish B6, from the turbid treatment, is shown with its section of spots used for the duration of the experiment, and the scale card is visible in the background. (Bottom) The section of spots as they move through the spot analysis process from ImageJ. From left to right: (1) Cropped image, (2) greyscale, (3) threshold adjusted, (4) spots analysed and numbered.

Each image is first length‐calibrated (in cm) using the ‘*Set scale’* command. The images were then cropped to focus on the spot patterns, converted to 8‐bit greyscale and enhanced by applying a bandpass filter (Fast Fourier Transform) to increase contrast. Images were then thresholded to produce binary images and low‐quality images were discarded at this point. Lighting conditions were constant in the aquarium, and therefore, a single threshold setting was applied to all experimental images. A polygon was drawn around the spot region of interest and the ‘*Analyze Particle*’ function was used to measure each spot. Measurement included spot area, perimeter, bounding rectangle (width & height), circularity and Fit ellipse (aspect ratio; Table S1). A region containing 5–10 spots was selected per individual, and the same spots were tracked across all time points. Spot area (cm^2^) was used as the measure of spot size.

#### 2.2.3 Statistical analysis

Six spot parameters were measured from each photograph across the five time points. The same spots were repeatedly measured on the same individuals, and therefore, the data are hierarchical (spots nested within individuals, nested within treatment) with repeated measures (time points) on the same spots. Linear mixed‐effects models (LMMs) were therefore used to test how each spot descriptor was affected by treatment and time. Each descriptor was modelled separately using the same structure: response ~ Treatment × factor(Time Point) + (1|Individual/Spot), where response was spot area or one of the other five shape descriptors. Treatment (clear or turbid) and time point were included as fixed effects, along with their interaction. Individual fish, and spot nested within individual, were included as random effects. Tank was initially included as an additional random effect; however, its variance was estimated at zero indicating negligible variation between tanks within a treatment and was therefore removed. The LMMs were conducted using the *‘lmer’* function (R package: ‘lme4’; Bates et al., 2015), with denominator degrees of freedom approximated using the Kenward‐Roger method (R package: ‘lmerTest’; Kuznetsova et al., 2017). Spot area, perimeter, width and aspect ratio were log‐transformed prior to analysis to meet the assumptions of normality and homoscedasticity. Height and circularity met these assumptions untransformed. Where the interaction (Treatment × Time point) was significant, we compared the model's estimated spot parameter at each time point against the baseline (Time point 1) for each treatment. This was to determine if there was a response to treatment (Time point 2–4) and if the response was reversed (Time point 5) when aquarium conditions were returned to standard, this was done using Holm correction for multiple comparisons (R package: ‘emmeans’; Lenth et al., 2019). For the log‐transformed responses, estimates were back‐transformed and are reported as percentage change relative to baseline (Time point 1). Prior to analysis, the TL of fish was compared between treatments to confirm that there was a size match.

### 2.3 Experiment 2: Estuarine comparison

#### 2.3.1 Field sampling

Spotted grunter were collected from the Breede Estuary (*n* = 76) in the Western Cape and the Kei Estuary (*n* = 63) in the Eastern Cape of South Africa using beach seine nets (Figure 3). The two estuaries differ markedly and characteristically in turbidity. In a regional physico‐chemical characterization of South African estuaries, average turbidity in the Kei was recorded at approximately 1300 NTU, compared with 7.4 NTU in the Breede (Harrison, 2004). This contrast was consistent with long‐term monitoring of the two systems (James et al., 2018; Nhleko, 2025) and was evident visually during sampling (Figure S2). The two systems also differ in temperature and salinity (Harrison, 2004), consistent with their different biogeographic locations. For each fish, the TL and fork length (FL) were recorded and a fin clip was taken. A full‐body photograph of the left, lateral side of each fish was captured against a black background with a scale card.

**FIGURE 3:**
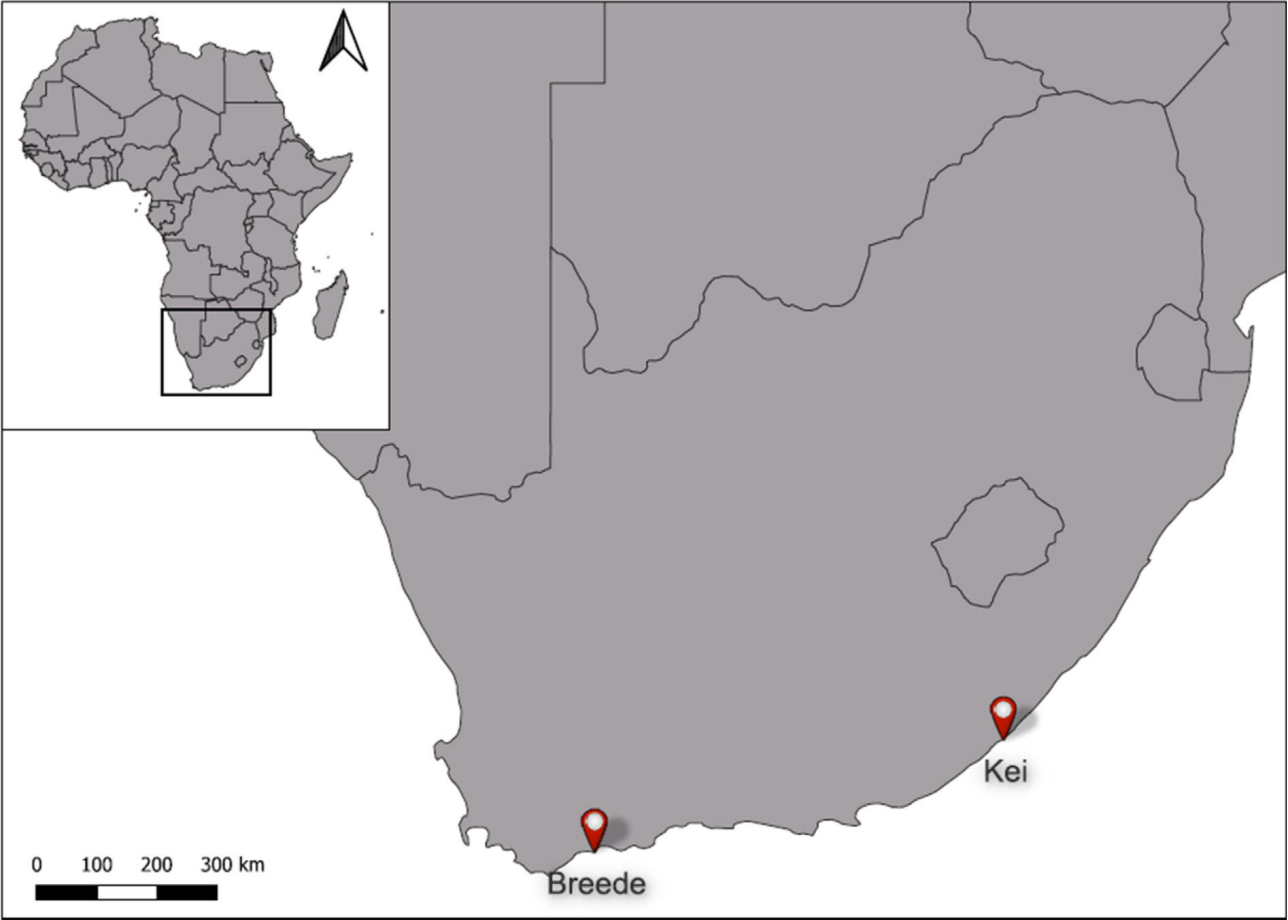
Study sites. Map of South Africa indicating the two study sites used for this study with the Breed Estuary in the Western Cape and the Kei Estuary in the Eastern Cape.

#### 2.3.2 Image analysis

Spot patterns of individuals were quantified using ImageJ (Schneider et al., 2012) following the same image‐enhancement pipeline as Experiment 1 (length calibration, 8‐bit greyscale conversion, bandpass filtering and thresholding to a binary image; Figure 2). However, because field images were captured under variable natural lighting, each image was thresholded individually so that the visible spots were accurately detected without introducing artefacts. The ‘Analyze Particle’ function was used to measure area, perimeter, bounding rectangle, fit ellipse, circularity and Feret's diameter for each spot (Table S1). In addition to the above process, the PAT‐GEOM plugin (Chan et al., 2019) which quantified three further shape properties: marking shape (Elliptical Fourier analysis with 20 harmonics), marking size (centroid size) and marking shape directionality (aspect ratio and orientation angle; Table S2; Chan et al., 2019).

#### 2.3.3 Statistical analysis

All statistical analyses were conducted in R (v.4.2.0) and R studio (R Core Team, 2021). All spot measurements were summarized to one value per individual so that the individual fish, rather than the spot, was the unit of replication. The size ranges of fish captured were not fully comparable, so the effect of estuary on each spot descriptor was assessed while controlling for body size. For the size‐dependent descriptors (spot number, area, perimeter, width, height, Feret diameter and centroid size), an analysis of covariance (ANCOVA) was conducted with estuary as a factor and FL as a covariate (descriptor ~ Estuary + FL). The assumption of homogenous slopes was tested for each ANCOVA (Estuary × FL interaction) and slopes were parallel for all size descriptors except spot number, for which the estuaries differed in the rate of spot accumulation with body size. Each size‐dependent descriptor was also compared between estuaries within a restricted, overlapping size range (FL 300–480 mm) where the two samples were directly comparable. For the dimensionless shape descriptors (circularity, aspect ratio and orientation angle), estuaries were compared directly using Wilcoxon rank sum tests. The *p*‐values from all spot descriptors were adjusted for multiple comparisons using the Holm correction. Residuals of the ANCOVA models were inspected and met the assumptions of normality and homoscedasticity.

Overall differences in spot patterning between the two estuaries were then examined using multivariate analyses on a consistent set of descriptors (spot number, perimeter, circularity, centroid size and aspect ratio). Variables were standardized (*z*‐scored) prior to analysis so that each contributed equally regardless of its measurement scale. A principal component analysis (PCA) was conducted to visualize spot pattern differences between the two estuaries using the R function ‘*prcomp*’. This was graphically displayed using the first two principal components. A permutational multivariate analysis of variance (PERMANOVA) calculated on Euclidean distances tested whether the two estuaries differed in overall spot patterns; this was conducted using the R function ‘adonis2’ with 9999 permutations (R package ‘vegan’; Oksanen et al., 2007). Since PERMANOVA can be sensitive to difference in multivariate dispersion between groups, the homogeneity of group dispersions was also tested (‘betadisper’). Finally, a linear discriminant analysis with leave‐one‐out cross‐validation (R package ‘MASS’; Ripley et al., 2013) was used to quantify how distinctly the two estuaries could be separated, expressed as the percentage of individuals correctly assigned to their estuary of origin and compared against the proportion expected by chance.

## 3 RESULTS

### 3.1 Experiment 1: Phenotypic plasticity

There was no significant difference in the TLs of fish from the two treatment groups (*W* = 74.5, *p* = 0.87), suggesting that any differences in spot patterning were not attributable to fish length.

#### 3.1.1 Spot number stability

Visual inspection revealed that spot number and position remained constant throughout the experiment for all individuals regardless of treatment. No spots disappeared and no new spots developed during the duration of the experiment (Figure S3).

#### 3.1.2 Spot‐size plasticity

Spot area was log‐transformed prior to analysis to meet model assumptions. The LMM showed that spot area did not differ between the two treatments overall (*F*_1,22_ = 0.24, *p* = 0.63), but it changed significantly over time (*F*_4,579.4_ = 56.89, *p* < 0.001) and more importantly the interaction between time and treatment was significant (*F*_4,579.4_ = 13.43, *p* < 0.001; Table 1). In the turbid treatment, spot area declined progressively below baseline, from approximately 24% smaller by time point 2 and around 30% smaller at time points 3 and 4 (*p* < 0.001; Table 2), but once these individuals were removed from the turbid treatment and returned to standard aquarium water conditions the spot area returned to within 6% of the baseline value (*p* = 0.12; Table 2, Figure 4). Conversely, fish from the clear treatment showed no comparable decline and instead fluctuated around the baseline for the first two time points with a slight decline of ~11% by time point 4. Once returned to standard aquarium conditions there was a 22% increase in spot size (*p* < 0.001). These results suggest that spotted grunter reduced their spot area in response to elevated turbidity and largely reversed this change when returned to clear conditions.

**TABLE 1:** Effect of turbidity and time on spot size.

Source of variation	SS	MS	Degrees of freedom		*F*‐value	*p*‐value
			Numerator	Denominator		
Treatment	0.011	0.011	1	22.0	0.24	0.629
Time point	10.233	2.558	4	579.4	56.89	<0.001
Treatment × time point	2.416	0.604	4	579.4	13.43	<0.001

*Note*: Results of the liner mixed‐effects model testing the effect of turbidity, time point and their interaction on the (log‐transformed) spot area of *Pomodasys commersonnii*. The sum of squares (SS), mean square (MS), Kenward‐Roger degrees of freedom, *F*‐value and *p* value are given for each.

**TABLE 2:** Comparing spot area at each time point relative to the baseline (Time point 1) within each treatment, from a linear mixed model fitted to a log‐transformed spot area.

Treatment	Contrast	Ratio	% change	SE	df	*t*‐value	*p*‐value
Clear	2 versus 1	0.957	−4.3	0.032	579	−1.32	0.375
	3 versus 1	0.984	−1.6	0.033	579	−0.48	0.862
	4 versus 1	0.892	−10.8	0.030	580	−3.39	0.002
	5 versus 1	1.223	+22.3	0.041	580	5.98	<0.001
Turbid	2versus 1	0.764	−23.6	0.028	579	−7.40	<0.001
	3 versus 1	0.698	−30.2	0.025	579	−9.88	<0.001
	4 versus 1	0.694	−30.6	0.025	579	−10.01	<0.001
	5versus 1	0.945	−5.5	0.034	579	−1.56	0.121

*Note*: Ratios are back‐transformed from the log scale (spot area at the time point relative to baseline); % change = (ratio – 1) × 100. *p* values are Holm‐adjusted.

**FIGURE 4:**
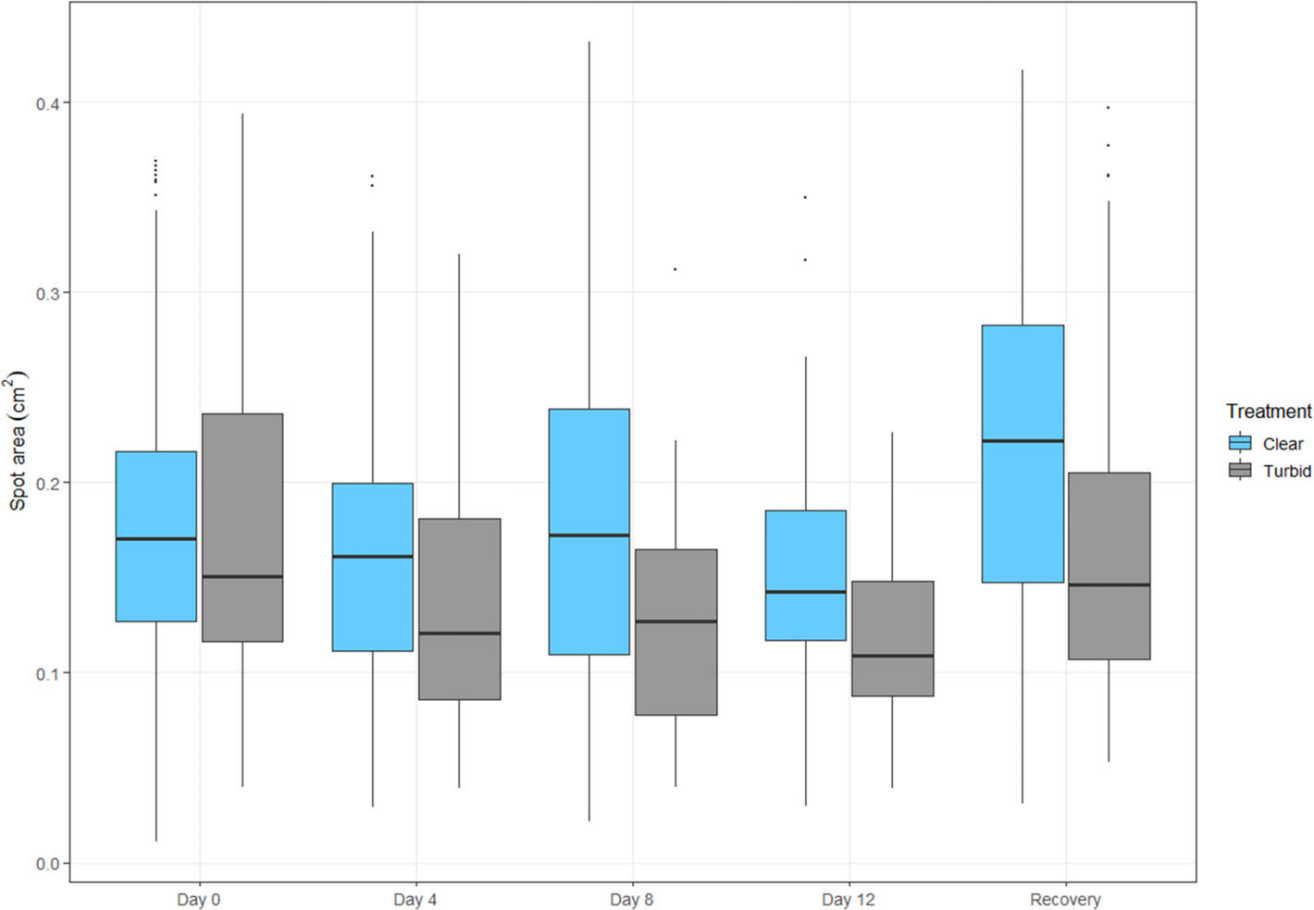
Spot area across the experiment. *Pomadasys commersonnii* spot area (cm^2^) in clear (blue) and turbid (grey) treatments across five time points. Recovery is after fish had been removed from treatment conditions. Statistical analysis was performed on log‐transformed spot area.

The same interaction was evident for three other size‐related descriptors: perimeter, width and height (Table 3) which indicates that turbidity altered spot size across multiple measures. Whereas the shape descriptors showed weaker responses to turbidity with no significant interaction for circularity (*p* = 0.087) and a weak interaction for aspect ratio (*p* = 0.020; Table 3). This weak significant interaction was not due to spot elongation during treatment exposure but rather a change occurring at time point 5, that is, post treatment; therefore, we conclude that turbidity has a reversible effect on spot size but not on spot shape.

**TABLE 3:** Turbidity effects on spot size and shape.

Spot descriptor	Treatment	Time point	Treatment × time point
Area	0.629	<0.001	<0.001
Perimeter	0.927	<0.001	0.008
Width	0.929	<0.001	<0.001
Height	0.201	<0.001	<0.001
Circularity	0.211	<0.001	0.087
Aspect ratio	0.014	0.020	0.020

*Note*: *p* values for each term from linear mixed models. Area, perimeter, width and aspect ratio were log‐transformed to meet model assumptions.

### 3.2 Experiment 2: Estuary comparison

There was a significant difference in the FLs of spotted grunter from the two estuaries with the Breede Estuary being composed of larger individuals (Mann–Whitney U; *W* = 3522, *p* < 0.001). Breede fish ranged from 298 to 615 mm (414.9 ± 89.35 FL, *n* = 76), and Kei fish ranged from 121 to 585 mm (332.4 ± 92.02, *n* = 63). Therefore, body size was accounted for during all comparisons between the two estuaries. Spot number increased significantly with FL in both estuaries, but the rate of this increase differed between estuaries (*F*_1,135_ = 4.00, *p* = 0.048; Figure 5). Breede fish accumulated spots more rapidly (0.39 spots/mm) with increasing fish size than Kei fish (0.29 spots/mm). Furthermore, where the two estuaries were directly comparable across overlapping size ranges, the fish from the Kei estuary had significantly fewer spots than those from the Breede (*p* < 0.001). Thus, after accounting for body size, fish from the more turbid estuary possessed fewer spots and accumulated spots slower during growth.

**FIGURE 5:**
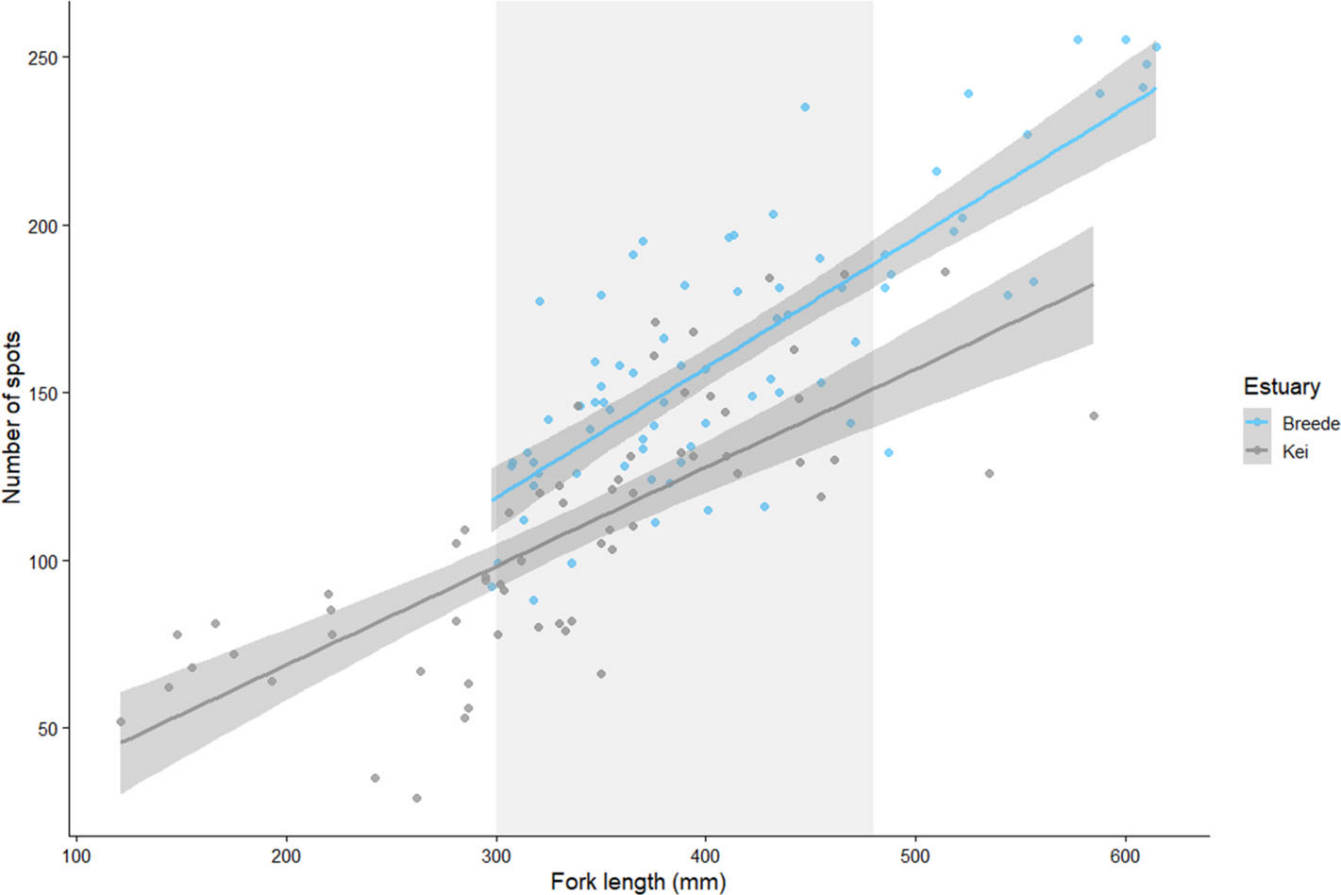
Relationship between fork length and number of spots for *Pomadasys commersonnii* from the Breede (blue) and Kei (grey) estuaries, with fitted linear regressions and 95% confidence intervals. The shaded band (fork length 300–480 mm) indicates the overlapping size range within which the two populations were directly compared.

In contrast, none of the spot‐size descriptors differed between estuaries once body size was taken into account (Table 4). For spot area, perimeter, width, height, Feret diameter and centroid size, FL was a strong predictor (*p* < 0.001) but estuary was not (*p* > 0.15). The restricted size range analysis reconfirmed this with no significant differences seen between estuaries for similar sized fish (*p* = 0.69). However, two of the three dimensionless shape descriptors showed a significant difference between estuaries. While circularity did not differ (*p* = 0.77), the aspect ratio and angle of orientation did. Kei spots were more elongated (aspect ratio 1.84 vs. 1.75; *p* < 0.001) and orientated differently (107° vs. 99.2°; *p* < 0.001) compared to Breede spots (Table 4).

**TABLE 4:** Univariate comparison of spot patterns between estuaries.

Spot descriptor	*p*‐value		
	Estuary		Fork length
	Original	Holm‐adjusted	
Size‐dependent descriptors (ANCOVA)			
Spot number	<0.001	<0.001	<0.001
Area	0.638	1.000	<0.001
Perimeter	0.948	1.000	<0.001
Width	0.153	1.000	<0.001
Height	0.295	1.000	<0.001
Feret diameter	0.696	1.000	<0.001
Centroid size	0.329	1.000	<0.001
Shape descriptors (Wilcoxon rank sum)			
Circularity	0.765	1.000	‐
Aspect ratio	<0.001	0.001	‐
Orientation angle	<0.001	<0.001	‐

*Note*: Size‐dependent descriptors were compared using ANCOVA while dimensionless shape descriptors were compared using Wilcoxon rank sum tests. The *p* values were Holm‐adjusted across comparisons.

Abbreviation: ANCOVA, analysis of covariance.

The multivariate analyses reflected the above results. The first two principal components explained a total of 82% of the variation in spot patterns with the two estuaries separated on both axes (Figure 6). PC1 represented overall spot size and number, loading positively on spot number, perimeter and centroid size, while PC2 (34%) represented spot shape, loading positively on aspect ratio and negatively on circularity. PERMANOVA confirmed that overall spot patterns differed significantly between estuaries (*R* ^2^ = 0.12, *F*_1,137_ = 19.35, *p* < 0.001). Group dispersions were also heterogeneous (*F*_1,137_ = 7.20, *p* = 0.008) with Kei fish showing greater variability in spot patterns compared to the Breede fish. The Linear Discriminant Analysis correctly assigned 83.5% of individuals to their estuary of origin (compared with 54.7% expected by chance: Breede 63/76 and Kei 53/63).

**FIGURE 6:**
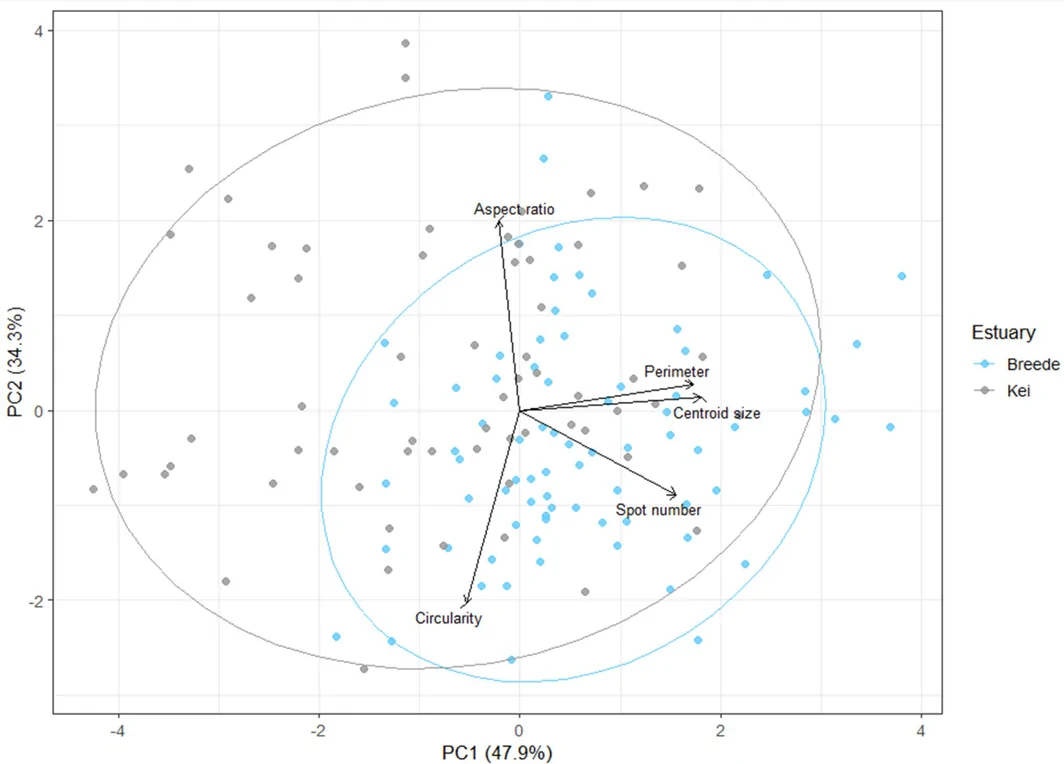
Principal component analysis of spot patterning. First two principal components of standardized spot descriptors for *Pomadasys commersonnii* from the Breede (blue) and Kei (grey) estuaries, with 95% confidence ellipses; each point is an individual fish.

## 4 DISCUSSION

This study provides the first evaluation of spot patterns in spotted grunter (*P. commersonnii*), demonstrating both phenotypic plasticity in response to turbidity and significant differences in spot patterns between fish from two estuaries with contrasting turbidities. The experimental component represents the first investigation of short‐term changes of spot patterns in this species and showed that spot number remained consistent regardless of turbidity level. This short‐term stability makes spot position suitable for individual identification (Merz et al., 2012), meeting the two key criteria for natural marking studies: (1) distinguishable between individuals and (2) temporal stability (Kristensen et al., 2020). Photo‐identification using natural markings has proven successful across diverse taxa including fish (McInnes et al., 2020), amphibians (Glimm et al., 2021; Mettouris et al., 2016), crustaceans and mammals (Nipko et al., 2020; Topličanec et al., 2022), offering a non‐invasive, low‐cost alternative to traditional tagging methods (Carpentier et al., 2016). Recent advances in computer vision and pattern recognition have further enhanced the reliability and scalability of these approaches (Crall et al., 2015).

However, the positive relationship between body size and spot number observed in both estuaries suggests that new spots develop as a fish grows. This is consistent with findings in Atlantic salmon (*Salmo salar*; Jørgensen et al., 2018; Stien et al., 2017) and trout (*Salmo trutta macrostigma*; Kocabas et al., 2011), where, although the number of spots increased throughout an individual's life, the initial spots from early life stages remained constant. This ontogenetic trait should thus be considered carefully when using spot patterns for individual re‐identification across extended time periods. Furthermore, the results show that an individual from the Breede estuary will have a greater number of spots compared to an individual from the Kei estuary of the same size, and the relationship between spot number and FL shows that fish from the Breede estuary develop their spots faster than those from the Kei estuary.

Spotted grunter exhibited significant plasticity in spot size in response to turbidity. Individuals in the turbid treatment presented with smaller spots than those in the clear treatment and this change was reversible when the fish were returned to lower turbidity levels. This suggests that this level of phenotypic plasticity is not developmental in nature but rather an acclimation/modulation process with continuous phenotypic variation.

This observed plasticity aligns with the sensory drive hypothesis, which suggests that signalling systems evolve to maximize transmission efficiency under local environmental conditions (Boughman, 2002; Endler, 1992). Sensory drive operates through both evolutionary adaptation and phenotypic plasticity, with individuals adjusting signal properties to match the local environment (Endler, 1992). In turbid, low‐visibility environments, it may not be energetically worthwhile to maintain larger, more conspicuous spot patterns; thus, this plasticity may function as both an energy‐saving tactic and a background‐matching response. The energetic costs of colour changes in some fishes have been established, with consequences for survival and higher food consumption (Bergstrom et al., 2012; Rodgers et al., 2013). These costs likely include the metabolic demands of pigment synthesis and reorganization, as well as resource allocation trade‐offs (DeWitt et al., 1998). Therefore, the reduction in spot conspicuousness observed in low‐visibility environments could be an adaptive response that minimizes energetic costs when the benefits of visual signalling are reduced (Van Buskirk & Steiner, 2009).

The size structure of spotted grunter differed between the two estuaries, with the Kei predominantly represented by smaller individuals. Spotted grunter are estuarine‐dependent marine spawners, and recruitment into any given estuary is strongly influenced by local spawning activity and estuary mouth dynamics (Butler et al., 2022; Childs et al., 2008). Notably, the Breede Estuary now supports a largely resident, self‐recruiting population that spawns within the vicinity of the system, whereas historically the species was thought to spawn only off KwaZulu‐Natal (Augustyn et al., 2017; Ziko et al., 2023). The size structure observed in each estuary is therefore likely to reflect these estuary‐specific recruitment and residency dynamics, rather than being a direct product of turbidity. However, the comparisons accounted for body size and so any differences we report would hold between fish of comparable size and are not an artefact of the differing size structures of the two populations.

Spotted grunter from the less turbid Breede estuary displayed more numerous spots compared to those from the turbid Kei estuary, even after accounting for body size. Spot number also increased with body size more steeply in Breede fish than in Kei fish, indicating that spots were not only more abundant but developed more rapidly during growth in the Breede estuary. In contrast to spot number, spot size did not differ between the estuaries once body size was taken into account, indicating that the between‐estuary differences were expressed in the number, and the shape, of spots rather than their size. Spots in the Kei estuary were more elongated and differently orientated compared to those in the Breede estuary. One possibility consistent with this pattern is developmental plasticity, in which early environmental conditions during critical developmental windows can have lasting effects on phenotype (West‐Eberhard, 2003). Under this interpretation, turbidity during the developmental programme could influence the emergence and accumulation of spots. Although there is no direct evidence indicating when spot ontogenesis begins in this species, other fish have been shown to develop spots between 197 and 230 days from conception (Merz et al., 2012), with ontogenetic colour changes serving both camouflage and communication functions across life stages (Booth, 1990). Juveniles of estuarine‐dependent species often occupy turbid environments, such as the Kei Estuary, where reduced visibility may provide a natural camouflage as they mature (Cyrus & Blaber, 1987). It is therefore possible that individuals in clearer systems, such as the Breede, develop and accumulate spots sooner, whether as a signalling or camouflage function. As disruptive colouration, these spots may break up body outlines and confuse potential observers (Duarte et al., 2017), while differences in spot shape and orientation between estuaries may further modify how the pattern is perceived. Alternatively, when in motion, the spots may create stripe‐like effects that facilitate shoal coordination (Price et al., 2008), an effect documented in other species that may serve as an anti‐predator mechanism by making speed and trajectory difficult to judge (Stevens, 2007).

The reversible reduction in spot size observed experimentally under turbid conditions is consistent with visibility‐based expectations. In high‐visibility environments, an individual's spots are detectable over a greater distance, so conspicuous spot patterning carries a higher detection cost where predators or competitors can see it from afar. In turbid, low‐visibility environments that cost is reduced and the benefit of maintaining large spots diminishes. A plastic, reversible reduction in spot size under turbidity is therefore consistent with a background‐matching or cost‐reducing response that tracks current visibility conditions, complementing the sensory drive interpretation above. The fact that spot size was reversible suggests that it reflects an acclimation mechanism tuned to present conditions rather than a permanent developmental outcome. This gradual, environmentally responsive patterning is consistent with the view that fish colour patterns can be shaped by environmental factors throughout ontogeny (Carr, 1969; Salis et al., 2019).

Taken together, the experimental and field results reveal that different components of the spot pattern respond to turbidity over different timescales. Spot size was labile and reversible: It changed within days under experimental turbidity and recovered when conditions cleared yet showed no standing difference between the estuaries once body size was accounted for. This apparent inconsistency is resolved by the reversibility itself. A trait that continuously recalibrates to present conditions is unlikely to accumulate into a fixed population‐level difference across estuaries where turbidity naturally fluctuates with tides, floods and season. Spot number behaved in the opposite way; it was stable over the short term of the experiment but increased with FL and differed between estuaries, indicating a cumulative, developmentally integrated trait that records the environment experienced during growth. Turbidity therefore appears to shape spot patterning through two distinct routes: a short‐term, reversible effect on spot size, and a longer‐term, developmentally accumulated effect on spot number. We note, however, that the absence of a field size difference could also reflect a smaller or less sustained natural turbidity contrast between the estuaries than that imposed experimentally, and further in‐depth studies in situ would help distinguish these possibilities.

Phenotypic plasticity is common in fish species and variation is often driven by genetic factors, environmental factors or a combination of the two (West‐Eberhard, 2003). The relative contributions of genetic versus environmental effects can vary across traits and populations, with some traits showing high heritability and others being predominantly plastic (Chevin & Lande, 2015). Understanding this balance is critical for predicting how populations will respond to environmental change (Donelson et al., 2018). In some fishes, such as brown trout (*Salmo trutta macrostigma*), spot patterns and colouration have a genetic basis (Kocabas et al., 2011). Aparicio et al. (2005) also found that three genotypes of brown trout had significant differences in body spot patterns. Whether the observed variation in spotted grunter spot patterns has a genetic basis is unknown; however, this possibility is important to consider for population studies and stock delineation.

Future research and improvements to this study could include: (1) examining spot ontogenesis from juvenile to adult stages to determine when and how spots develop; (2) measuring spot colouration repeatedly over time and across estuaries and environments to test predictions about pigmentation intensity; this may also serve as a useful marker for environmental change, particularly with increasing pollution in estuaries; (3) expanding sampling to include additional estuaries across the species' range and (4) conducting experiments to disentangle genetic adaptation from phenotypic plasticity and determine whether spot pattern differences have a heritable basis.

The limitation of this study was the high number of individuals with skin abrasions from the seine net. The use of less invasive capture methods that better preserve skin integrity is therefore encouraged. Additionally, low‐quality photos remain a challenge for image analysis studies as variation in angle, lighting and camera quality can all influence the accuracy of the results (Merz et al., 2012).

In conclusion, this study demonstrates that spotted grunter exhibit both short‐term phenotypic plasticity and local environmental variation in spot patterns associated with turbidity. Spot number and position remain stable over the short term, allowing reliable individual identification, while spot size and colouration display reversible plasticity. Estuarine differences were expressed in the number and shape of spots rather than their size: Fish from the less turbid Breede estuary had more numerous spots and developed them rapidly with growth, compared to fish from the turbid Kei estuary. These findings support the sensory drive hypothesis (Boughman, 2002; Endler, 1992) and highlight the important role of environmental conditions, particularly turbidity, in shaping phenotypic variation in this species. The reversible nature of spot size suggests a physiological mechanism operating on a short timescale, whereas the between‐estuary differences in spot number more likely reflect developmental plasticity or local adaptation accumulated over ontogeny. The observed plasticity demonstrates how organisms balance multiple selective pressures through flexible phenotypic responses (DeWitt et al., 1998). The high degree of spot plasticity observed in size suggests caution when using spot patterns alone for stock discrimination in *P. commersonnii*, as turbidity‐driven phenotypic variation could be misinterpreted as genetic differentiation between populations. However, the more stable differences in spot number and shape may serve as more reliable population markers, although whether they reflect developmental plasticity or genetic differentiation needs to be established. The short‐term temporal stability of spot number and position nonetheless makes them suitable for individual re‐identification studies and mark‐recapture programmes with the acknowledgement that more spots will be added as an individual grows. The quantitative framework developed here provides a foundation for future research into the adaptive significance and genetic basis of colour pattern variation in spotted grunter.

## AUTHOR CONTRIBUTIONS

All authors contributed to the conception and design of the study and participated in field sampling. Vivienne Coetzee and Denham Parker conducted the aquarium experiment. Vivienne Coetzee analysed the data and performed the statistical analyses. The main manuscript was prepared by Vivienne Coetzee and all authors reviewed and edited the final version.

## FUNDING INFORMATION

This research was supported by the Foundational Biodiversity Information Programme (FBIP), funded by the Department of Science and Innovation (DSI) and managed by the South African National Biodiversity Institute (SANBI) and the National Research Foundation (NRF) (Grant No 128330).

## Supporting information


**Figure S1.** Photographs of treatment tanks: (a) Clear control tank and (b) turbid treatment tank. (c) The turbidity level of each tank was measured throughout the experiment. The black bars on top represent the tank number.
**Table S1:** ImageJ ‘Particle Analysis’ Measurements used to analyse the spot patterns of a fish species: *Pomadasys commersonnii*. A description and depiction are given for each measurement.
**Table S2:** PAT‐GEOM measurements selected for spot pattern analysis of *Pomadasys commersonnii*. PAT‐GEOM is a free open‐source ImageJ plugin.
**Figure S2:** Study Sites: Photographs highlighting the contrast in turbidity between two South African estuaries: Breede Estuary (left) and the Kei Estuary (right).
**Figure S3**. Spots over time: Spots (1–8) from a *Pomadasys commersonnii* (B6) exposed to high turbidity levels over time. (Top: left–right) Replicate one (with the over‐lying spot numbers). Replicate two and replicate three. (Bottom: left–right) Replicate four, replicate five and the particle analysis outline produced by ImageJ shows spot numbers.

## Data Availability

The data that support the findings of this study are available upon reasonable request. Please email the corresponding author.
