# Supplementary material for: Spot the difference: Turbidity‐driven plasticity and variation in spotted grunter (Pomadasys commersonnii) spot patterns
Source: J Fish Biol. 2026 Sep 10;109(5):2709–21. doi: 10.1111/jfb.70646 (PMC13770689; doi:10.1111/jfb.70646)
Supplement: Supplementary file 1 [file JFB-109-2709-s001.docx]

Supplementary Information

**Supplementary Fig 1. Photographs of treatment tanks:** (a) Clear control tank and (b) turbid treatment tank. (c) The turbidity level of each tank was measured throughout the experiment. The black bars on top represent the tank number.

| **Supplementary Table 1: ImageJ ‘Particle Analysis’ Measurements** used to analyse the spot patterns of a fish species: *Pomadasys commersonnii*. A description and depiction are given for each measurement. | | |
| --- | --- | --- |
| **Measurement** | **Description** | **Diagram** |
| Area | The area of each spot is calculated in cm^2^ |  |
| Perimeter | The length of the outside boundary of each spot is calculated in centimetres. |  |
| Bounding rectangle | There are two measurements taken for this measurement: Width and Height. This is the measurement of the smallest rectangle enclosing the selection. |  |
| Fit Ellipse | The best fitting ellipse to each spot. Measurements given include the primary (Major) and secondary (Minor) axis of the best-fitting ellipse. |  |
| Circularity | A value to indicate the circularity of each spot where a perfect circle will have a value of 1 and elongated shapes will have values closer to 0.  Equation 2.2:  $Circularity=4\pi\frac{Area}{{Perimeter}^{2}}$ |  |
| Ferret’s Diameter | The longest distance between any two points on the spot boundary. |  |

| **Supplementary Table 2: PAT-GEOM measurements** selected for spot pattern analysis of *Pomadasys commersonnii*. PAT-GEOM is a free open-source ImageJ plugin. | | |
| --- | --- | --- |
| **Measure** | **Description** | **Depiction** |
| Marking shape – Elliptical Fourier analysis | Approximates the outline of each spot using a specified number (20) of harmonics. Each harmonic describes the shape of the marking using a numerical descriptor. The greater the number of harmonics (n), the more detailed the outline. |  |
| Marking shape Directionality | Aspect ratio: calculated by dividing the length of the minor axis (blue) by the length of the major axis (red).  Orientation (θ): a measure of the angle between the major axis and a horizontal line(white) across the spot. |  |
| Centroid size | Average centroid size of each spot. This is calculated using the distance (d) from the centroid to the boundary of each spot by a given number of points (N).  Equation 2.3:  $=\frac{1}{N}\sqrt{\sum_{i=1}^{N} d_{i}^{2}}$ |  |

**Supplementary Figure 2: Study Sites:** Photographs highlighting the contrast in turbidity between two South African estuaries: Breede Estuary (left) and the Kei Estuary (right).

**Supplementary Figure 3. Spots over time**: Spots (1-8) from a *Pomadasys commersonnii* (B6) exposed to high turbidity levels over time. (Top: left-right) Replicate one (with the over-lying spot numbers), Replicate two and replicate three. (Bottom: left–right) Replicate four, replicate five and the particle analysis outline produced by ImageJ shows spot numbers.
